# A heterodimer of hemoglobin identifies theranostic targets on brain‐metastasizing melanoma cells

**DOI:** 10.1002/ijc.35458

**Published:** 2025-04-26

**Authors:** Maharrish Chelladurai, Orit Sagi‐Assif, Shlomit Ben‐Menachem, Tsipi Meshel, Metsada Pasmanik‐Chor, Sivan Izraely, Dave S. B. Hoon, Isaac P. Witz

**Affiliations:** ^1^ The Shmunis School of Biomedicine and Cancer Research George S. Wise Faculty of Life Science, Tel Aviv University Tel Aviv Israel; ^2^ Bioinformatics Unit The George S. Wise Faculty of Life Science, Tel Aviv University Tel‐Aviv Israel; ^3^ Department of Translational Molecular Medicine and Sequencing Center Saint John's Cancer Institute at Providence Saint John's Health Center Santa Monica California USA

**Keywords:** JQ1 (BRD4 inhibitor), melanoma, melanoma brain metastasis, NT157 (IRS2 inhibitor), tumor microenvironment

## Abstract

Cancer microenvironments encompass both cancer‐promoting and cancer‐restraining factors. If these factors cancel each other, cancer dormancy may ensue. In search of microenvironmental factors that keep dormant lung‐metastasizing neuroblastoma cells and brain‐metastasizing melanoma cells (BMMC) in check, we identified the beta subunit of hemoglobin and a heterodimer of alpha and beta chains of hemoglobin (α/β dimer) in the lung and brain microenvironments, respectively, as anti‐metastatic factors. A previous study demonstrated that the α/β dimer triggers programmed cell death of BMMC and downregulates the expression of BRD4, GAB2, and IRS2 proteins, which perform essential functions in tumorigenesis and progression. The working hypothesis of the present study is that in addition to its tumoricidal function, the α/β dimer serves as a pathfinder for the identification of therapy targets for BMMC. We, therefore, employed small‐molecule inhibitors of Bromodomain‐containing protein 4 (BRD4), GRB2‐associated‐binding protein 2 (GAB2), and Insulin receptor substrate 2 (IRS2) as potential anti‐BMMC agents. A combination of sub‐lethal concentrations of BRD4 and IRS2 inhibitors synergistically arrested BMMC at the subG1 phase of the cell cycle and killed more than 70% of BMMCs. The BRD4/IRS2 inhibitor cocktail (designated hereafter as BRIRi) moderated the malignancy of BMMC lines from four different human melanomas. Preliminary results suggest that the BRIRi modulated “cold” BMMC to “hot” ones. Among the top enriched functions of differentially expressed genes identified by RNAseq of BRIRi‐treated versus control BMMC, TNF and apoptotic signaling pathways were observed. We propose that co‐targeting BRD4 and IRS2 offers a promising approach for treating BMMC.

## Graphical abstract

What's new?

Hemoglobin‐derived molecules have been shown to exhibit innate anti‐cancer functions similar to antimicrobial peptides and proteins. Previously, the authors identified a heterodimer of the alpha and beta chains of hemoglobin in the brain microenvironment that killed brain‐metastasizing melanoma cells by targeting BRD4 and IRS2 proteins. Here, they found that small‐molecule inhibitors of BRD4 and IRS2, in combination, mimicked the effects of the hemoglobin alpha/beta heterodimer and restricted the malignant phenotype of brain‐metastasizing melanoma cells. Taken together, the findings highlight the potential of innate anticancer factors to help identify therapy targets on cancer cells.

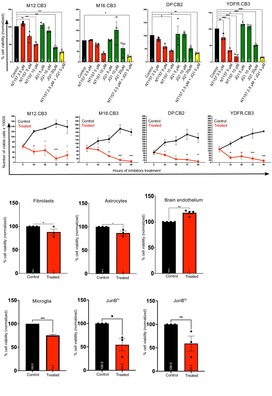

**Abbreviations**
BEC: brain endothelial cells
BMMC: brain‐metastasizing melanoma cells
BRD4: bromodomain containing 4
BRIRi: brd4 and irs2 inhibitors
CCL: Chemokine (C‐C motif) ligand
DEG: differentially expressed genes
FACS: fluorescence‐activated cell sorting
FCS: fetal calf serum
GAB2: grb2 associated binding protein 2
GAPDH: glyceraldehyde 3‐phosphate dehydrogenase
HBB: beta subunit of hemoglobin
HSP70: Heat Shock Protein 70
IRS2: insulin receptor substrate 2
PARP: poly (adp‐ribose) polymerase
PD‐L1: programmed death‐ligand 1
PI: propidium iodide
TME: tumor microenvironment
TNF: tumor necrosis factor

## 1 INTRODUCTION

Two major opposing forces function in the microenvironment of most tumors. One blocks cancer progression mainly via the host immune system,^1^, ^2^ while the other promotes cancer progression by various stromal non‐cancerous cells.^3^ When these two opposing vectors cancel each other and create a balanced equilibrium, cancer cells may enter a state of dormancy.

Early studies indicated that in addition to immune surveillance against cancer, other mechanisms, such as intercellular contact, could suppress cancer outgrowth.^4^, ^5^ Based on these early studies, Klein proposed a general, non‐immunological mechanism termed “intercellular surveillance,”^6^ The intercellular surveillance or microenvironmental control concept postulates that nascent cancer cells are killed, growth‐arrested, or induced to express a non‐malignant phenotype via contact with neighboring non‐cancerous cells.^6^

Previous studies from our group expanded the concept of intercellular surveillance to include organ‐specific soluble factors that keep dormant micrometastatic cancer cells in check.^7^ In a previous study, we identified the beta chain of hemoglobin produced by alveolar endothelial and epithelial cells as an innate anti‐metastatic factor mediating growth arrest and apoptosis of lung‐metastasizing neuroblastoma cells.^8^

In studies on the cross‐talk between brain‐metastasizing melanoma cells (BMMC) and the metastatic microenvironment of the brain, we recently discovered that a brain‐derived hemoglobin heterodimer composed of alpha and beta chains mediates growth arrest, apoptosis, and necrosis of BMMC.^9^

Moreover, the dimer downregulates the expression of Bromodomain‐containing protein 4 (BRD4), GRB2‐associated‐binding protein 2 (GAB2), and Insulin receptor substrate 2 (IRS2) proteins, all of which play crucial roles in cancer cell sustainability and progression.^10^, ^11^, ^12^

The working hypothesis of this study is that in addition to its anti‐melanoma functions, the dimer can be employed as a pathfinder for possible theranostic targets on progressing metastatic melanoma cells. Drugs that target such molecules may be employed as anti‐cancer drugs. Indeed, published work indicates that the inhibition of BRD4, GAB2, or IRS2 restrains the progression of several types of cancer.^11^, ^13^, ^14^

In the present study, we found that small‐molecule inhibitors of BRD4 and IRS2, in combination, mimicked the effects of the hemoglobin alpha/beta heterodimer and restricted the malignant phenotype of BMMC.

## 2 MATERIALS AND METHODS

### 2.1 Cell culture

Brain‐metastasizing melanoma cell (BMMC) line YDFR.CB3 was established from the parental cell line YDFR (kindly provided by Prof. Michael Micksche, Department of Applied 3 and Experimental Oncology, Vienna University, Austria). Likewise, other BMMC lines DP.CB2, M12.CB3, and M16.CB3 were established from the parental cell lines DP‐0574 Me, UCLA‐SO‐M12, and UCLA‐SO‐M16 (kindly provided by Prof. Dave S.B. Hoon), respectively (RRID:CVCL_5T78).^15^, ^16^ In short, human brain metastatic melanoma cells were inoculated subdermally into nude mice to establish the cutaneous (C) variants. Cells from these cultures were inoculated intracardially and passaged in the brain for two or three passages, yielding the brain metastatic variants (CB2 or CB3, respectively). Melanoma cells, including both brain metastatic variants and cutaneous variants, were maintained in RPMI‐1640 medium (Sartorius, Kibbutz Beit Haemek, Israel) supplemented with 10% fetal calf serum (FCS), 2 mmol/mL L‐glutamine, 100 units/mL penicillin, 0.1 mg/mL streptomycin, and 12.5 units/mL nystatin. Immortalized human microglia‐SV40 cells (ABM, Milton, Canada) were maintained on plates coated with 100 μg/mL collagen I, rat tail (Corning, Bedford, MA) in Prigrow III medium (ABM) supplemented with 10% FCS. Microglia knocked in and knocked out with JUNB were generated and maintained, as mentioned previously.^17^ Human brain endothelial cells (hCMECs/D3 cells) (RRID:CVCL_U985) were maintained in monolayer cultures as previously described.^18^ Human astrocytes (HAs; ScienCell Research Laboratories, USA) were maintained on 0.1% poly‐L‐lysine (Sigma‐Aldrich, St. Louis, MO) in an astrocyte growth medium (ScienCell Research Laboratories). Human dermal fibroblasts (kindly provided by Prof. Carmit Levy, Sackler School of Medicine, Tel Aviv University) were cultured in RPMI supplemented with 10% FBS. 0.5% FCS‐supplemented medium was used for inhibitor suspension and starvation in all the experiments. BMMC lines expressing mCherry were transduced with a pQCXIP‐mCherry plasmid (Clontech Laboratories, Inc.), as previously described.^16^, ^19^ All experiments were performed with mycoplasma‐free cells. Cells were cultured in an incubator with humidified air with 5% CO2 at 37°C. All human cell lines have been authenticated using STR profiling within the last 3 years.

### 2.2 Viability assay (XTT)

Four Cancer cells were seeded at a density of 5000 cells per well in a 96‐well plate and incubated overnight. Varying concentrations of NT157 (IRS2 inhibitor) (Selleckchem, Houston, Texas, USA), (+)‐JQ1 (BRD4 inhibitor) (Sigma‐Aldrich), and Imperatorin (GAB2 inhibitor, Sigma Aldrich) were used to determine their cytotoxicity. Cells were treated with inhibitors for 24 h. Starvation medium containing 0.001% DMSO was used as a vehicle control for all the experiments. Cell viability was measured using Cell Counting Kit‐8 XTT (Sigma‐Aldrich) according to the manufacturer's instructions.

### 2.3 Cell cycle analysis

5 × 10^5^ BMMC were plated overnight in 24 well plates and treated with the inhibitors (2.5 μM of NT157 and 5 μM of JQ1) or starvation medium with 0.001% DMSO control alone for 24 h and pelleted. Cell pellets were washed and resuspended in 0.5 mL PBS containing 0.05 mL propidium iodide (PI) (50 mg/mL) (Sigma‐Aldrich) and 0.05 mL Triton X‐100. Cells were incubated for 10 m in the dark. Flow cytometry analysis was performed using an S100EXi flow cytometer (Stratedigm, Inc., San Jose, CA). The raw data was analyzed using FlowJo v10 RRID:SCR_008520 (FlowJo, Ashland, OR). The final figure was created using Inkscape.

### 2.4 Annexin V‐FITC/PI apoptosis assay

5 × 104 melanoma cells were cultured overnight in a 24‐well plate. Cells were treated with the inhibitors (2.5 μM of NT157 and 5 μM of JQ1) or starvation medium with 0.001% DMSO control for 24 h. The percentage of cells in early apoptosis, late apoptosis, and necrosis was measured using MEBCYTO Apoptosis Kit (Annexin V‐FITC Kit) according to the manufacturer's instructions. Flow cytometry analysis was performed using an S100EXi flow cytometer (Stratedigm). Raw data for the FACS plot were analyzed using Flowjo V10 software RRID:SCR_008520. The percentage of dead and live cells was determined. FITC logH and PI logH were expressed as X and Y axes, respectively. A quadrant was placed in a way that contained more than 95% of live cells in the control sample. The same quadrant was applied to the BRIRi treated sample.

### 2.5 Growth curve

5 × 10^5^ melanoma cells were cultured overnight in a 60 mm plate. Cells were treated with 2.5 μM of NT157 and 5 μM of JQ1 for 0, 24, 48, 72, and 96 h. Inhibitors, suspended in a starvation medium, were added once at the beginning of each time point. After each time point, cells were trypsinized and counted in a hemocytometer after staining with trypan blue.

### 2.6 Migration through extracellular matrix

6 × 10^5^ melanoma cells were plated in a 60 mm plate overnight. Melanoma cells were then treated with starvation medium with 0.001% DMSO or inhibitors for 3 h, counted, and 1 × 10^5^ treated cells were loaded onto collagen‐coated transwell inserts (8 μm; Corning Costar Corp., New York, NY) and allowed to migrate for 24 h toward 10% FCS medium. Then, the upper side of the transwell chamber was scraped gently with cotton swabs to remove non‐migrating cells. Cells at the bottom side of the transwell inserts were fixed with ice‐cold methanol for 5 m. Migrating cells were stained with a Dif‐stain Kit (Kaltek, Padova, Italy) according to the manufacturer's instructions. The number of melanoma cells transmigrating to the underside of the membrane was determined in each experiment by counting at least six independent fields in duplicates using an Olympus IX53 inverted microscope (Olympus, Center Valley, PA).

### 2.7 Transendothelial migration through a BBB model

Transwell inserts with a pore size of 8 μm were coated with collagen I, rat tail (Corning, Bedford, MA) for 1 h at 37°C. Brain endothelial cells (BECs) were loaded onto the upper side of the apical chamber and allowed to create a monolayer for 48 h. mCherry‐expressing melanoma cells (treated with starvation medium with 0.001% DMSO or inhibitors) were then loaded onto the BEC monolayer and allowed to migrate for 24 h toward a 10% FCS‐supplemented medium. At the end of the incubation period, the upper side of the apical chamber was scraped gently with a cotton swab to remove nonmigrating cells. The membranes were fixed with 4% paraformaldehyde, washed with PBS, and mounted with Dapi Fluoromount‐G (Southern Biotech, Birmingham, AL, USA). The number of melanoma cells transmigrating to the underside of the membrane was determined by counting five high‐power fields under fluorescence microscopy in duplicates (Nikon Eclipse TE 2000‐S, Nikon).

### 2.8 Wound‐healing assay

Melanoma cells were seeded on a collagen‐coated 96‐well plate. Upon confluency, cells were treated with a starvation medium with 0.001% DMSO or inhibitors for 3 h. Cells were washed with PBS twice, and a fresh starvation medium was added. Then, the cell monolayer was scratched using a 96‐well WoundMaker (Sartorius, Gottingen, Germany). Cells were washed twice, and a starvation medium was added to the cells for 24 h. The wounds were imaged every 3 h for 24 h, and images were analyzed using the IncuCyte S3 system (Sartorius). Each experiment was repeated at least three times.

### 2.9 Adhesion to brain endothelial cells

Adhesion of melanoma cells to BECs was performed as described previously.^19^ In brief, 5 × 10^4^ hCMEC/D3 cells were cultured for 24 h to form a confluent monolayer on 96‐well cell culture plates coated with Collagen I. 1 × 10^5^ mCherry‐labeled melanoma cells, treated for 3 h with starvation medium with 0.001% DMSO or inhibitors, were seeded onto the BECs. After 24 h, the fluorescence of mCherry‐expressing cells was measured at a wavelength of 590/645. Cells were then washed twice with PBS to remove non‐adherent cells, and the fluorescence of mCherry‐expressing adherent cells was measured again at a wavelength of 590/645. To obtain the cell relative adhesion capacity, the OD of the adherent cells after washing with PBS was divided by the OD of the total cells before washing with PBS.

### 2.10 Western blot

1.34 ×10^6^ cells were plated in a 100 mm cell culture plate overnight, then treated with the inhibitors or starvation medium with 0.001% DMSO for 24 h. Floating cells were collected and pelleted by centrifugation. Similarly, the adhered cells were pelleted after trypsinization. Protein detection by Western blot was performed as previously described,^20^ where 30 μg of cell lysate was loaded. Primary antibodies against the following proteins were used: Poly (ADP‐ribose) polymerase (PARP) (Cell Signaling Technology Cat# 9542, RRID:AB_2160739), cleaved caspase 7 (Cell Signaling Technology Cat# 9491, RRID:AB_2068144), Heat Shock Protein 70 (HSP70) (Cell Signaling Technology Cat#4872, RRID:AB_2279841), MYC (Cell Signaling Technology Cat# 9402, RRID:AB_2151827), and Glyceraldehyde 3‐phosphate dehydrogenase (GAPDH) (Cell Signaling Technology Cat# 5014, RRID:AB_10693448). Horseradish peroxidase‐conjugated goat anti‐mouse (Jackson ImmunoResearch Labs Cat# 115–035‐003, RRID:AB_10015289) or goat anti‐rabbit (Jackson ImmunoResearch Labs Cat# 111–035‐003, RRID:AB_2313567) were used as secondary antibodies. The bands were visualized by chemiluminescence ECL reactions (Merck Millipore, Darmstadt, Germany) and imaged using The ImageQuant 800 system (GE Healthcare, Chicago, IL). Band density was quantified using ImageJ.

### 2.11 PD‐L1 expression

6 × 10^5^ melanoma cells were plated in a 60 mm plate overnight. Cells were then treated with a starvation medium with 0.001% DMSO or inhibitors for three h, washed with PBS, and starved for 24 h. Cells were then pelleted and incubated with an anti‐PD‐L1 antibody (Ab) (Biolegend Cat# 393608, RRID: AB_2749925 [BioLegend Cat. No. 393608]) for 45 m in the dark. After a brief wash with PBS, cells were incubated with a FITC‐conjugated secondary Ab (Biolegend) for 45 m. Flow cytometry analysis was performed using an S100EXi flow cytometer and FlowJo v10 RRID:SCR_008520. The raw data was analyzed using FlowJo v10 RRID:SCR_008520. Dead cells were gated out from the analysis. The percentage of cells positive for PD‐L1 was calculated by subtracting cells positive for PD‐L1 Ab from the secondary Ab control. The final image was created using Inkscape.

### 2.12 RNA preparation and reverse transcription quantitative real‐time PCR (RT‐qPCR)

Total cellular RNA was extracted using Tri Reagent (Sigma‐Aldrich). The RNA concentration was determined with nanodrop absorbance at 260 nm (A260) and continued eight with downstream applications only if the absorbance was A260/A280 = 1.8–2.1. RNA samples were used for cDNA synthesis using the qScript cDNA Synthesis Kit (Quantabio, Beverly, MA) according to the manufacturer's instructions. Amplification reactions were performed with SYBR Green I (Thermo Fisher Scientific, Rochester, NY) in duplicates in a Bio‐Rad CFX Connect Real‐Time PCR Detection System HTqPCR (RRID:SCR_003375) (Bio‐Rad Laboratories, Hercules, CA). PCR amplification was performed for 40 cycles (95°C for 15 s, 59°C for 20 s, and 72°C for 15 s). The following primers were used: CCL2 forward‐TGCAATCAATGCCCCAGTCAC, CCL2reverse‐ACTTCTGCTTGGG GTCAGCAC, CCL3 forward‐ATGGCTCTCTGCAACCAGTTCT, CCL3 reverse‐CGCTTGGTTAGGAAGATGACAC, CCL4 forward‐CAATGGGCTCAGACCCTCCC, CCL4 reverse‐AGGATTCACTGGGATCAGCACA, RS9 forward‐CGGAGACC CTTCGAGAAATCT, RS9 reverse‐GCCCATACTCGCCGATCA.

### 2.13 RNA sequencing (RNA‐seq) analysis

The four BMMC lines were seeded at 6 × 10^5^ in a 60 mm plate overnight. Cells were treated with 2.5 μM of NT157, 5 μM of JQ1, or with a combination of both 2.5 μM of NT157 and 5 μM of JQ1 for 3 h. RNA extraction and sequencing were performed as previously described.^20^ The sequencing coverage and quality statistics for each sample are summarized in Table S1. Raw RNA‐seq reads were checked for overall quality and filtered for adapter contamination using Trimmomatic (version 0.36) RRID:SCR_011848.^21^ The filtered reads were then mapped to the GENCODE RRID:SCR_004463 comprehensive gene annotation reference set (version 19) using the STAR aligner (version 2.4.2a) RRID:SCR_004463^22^ with default parameters. Read counts for each feature were generated using the “quantModeGeneCounts” function in STAR. Significantly differentially expressed genes (DEGs) were identified using ANOVA with a significance cutoff pAdj <0.05 and fold change (FC) FC ≤ −2 or FC ≥2. Enriched biological processes of the differentially expressed genes were obtained through the Database for Annotation, Visualization, and Integrated Discovery (DAVID) DAVID (RRID:SCR_001881).^23^, ^24^ The Venny tool v2.1 (https://bioinfogp.cnb.csic.es/tools/venny/index.html) was used to compare between differentially expressed gene lists.

### 2.14 Biostatistical analysis

Data were analyzed using Student's t‐test and considered significant at *p* values ≤0.05. *p < 0.05, **p < 0.01, ***p < 0.005, ****p < 0.001. Bar graphs represent the mean and standard error of the mean (SEM) across multiple independent experimental repeats. Experiments were repeated at least three independent times. Graphs were generated using GraphPad Prism 9 GraphPad Prism (RRID:SCR_002798). Inkscape V1.1 and ImageJ RRID:SCR_003070 were used as the final image editing software.

## 3 RESULTS

### 3.1 The co‐targeting of BRD4 and IRS2 induces growth arrest and apoptosis in brain‐metastasizing melanoma cells (BMMC)

JQ1 and NT157 are small‐molecule inhibitors targeting BRD4 and IRS2, respectively.^25^, ^26^ Imperatorin is a small molecule inhibitor of GAB2 phosphorylation and the downstream MAPK, PI3K/AKT, and NF‐κB signaling pathways.^27^ Initial dose–response cytotoxicity assays revealed that even high concentrations of imperatorin were relatively inactive in killing BMMC (Figure S1). We, therefore, did not use this inhibitor in subsequent experiments. The dose–response assays demonstrated that treating BMMC with 10 μM of NT157 or with 20 μM of JQ1 inhibited approximately 50% of cell viability. 2.5 μM of NT157 or 5 μM of JQ1 for 24 h did not reduce BMMC viability.

**FIGURE 1:**
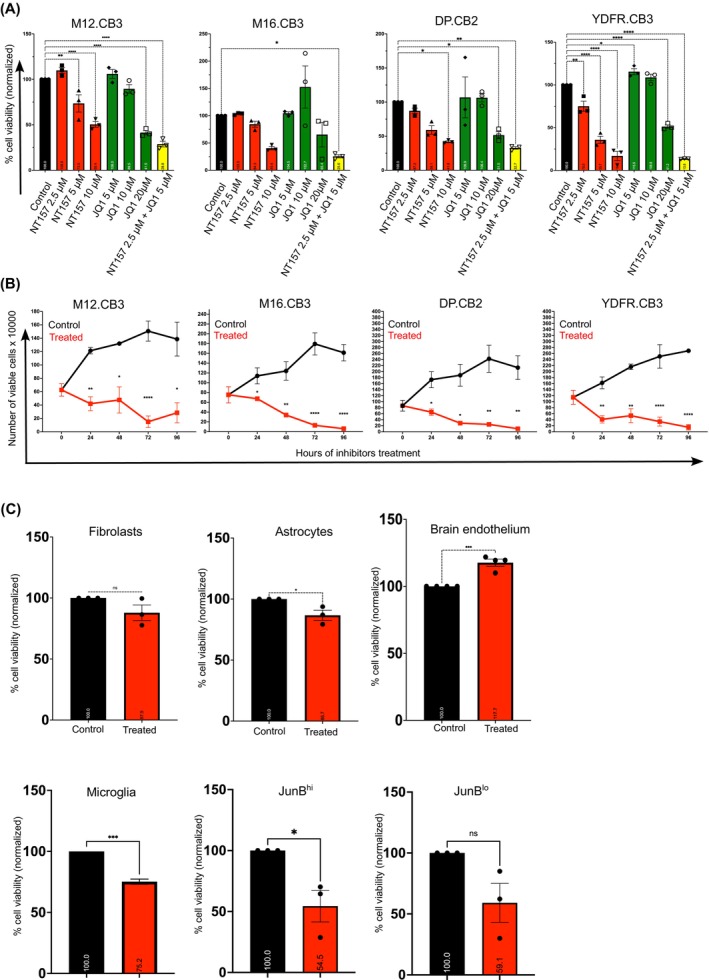
BRD4 and IRS2 inhibitors target both melanoma and melanoma‐promoting non‐cancerous microenvironmental cells. (A) Cytotoxicity of NT157 (IRS2 inhibitor) and JQ1 (BRD4 inhibitor) on four BMMC was evaluated using XTT assay after treating cells for 24 h. Starvation medium (0.5% FCS) with 0.001% DMSO served as a control. Results are shown as mean ± SEM of three independent experiments. Significance was evaluated using one‐way ANOVA. *p < 0.05, **p < 0.01, ****p < 0.001. (B) The growth curve of the four BMMCs was obtained by treating them once with BRIRi and quantifying viable cells manually using trypan blue every 24 h up to 96 h. Results are shown as mean ± SEM of three independent experiments. Significance was evaluated using the Student's *t* test. *p < 0.05, **p < 0.01, ****p < 0.001. (C) Cytotoxicity of BRIRi on non‐cancerous cell lines such as fibroblasts, astrocytes, brain endothelial cell line, microglia, microglia‐JunB^hi^, and microglia‐JunB^lo^ was evaluated after treatment with BRIRi for 24 h. Results are shown as mean ± SEM of three independent experiments. Significance was evaluated using the Student's *t* test. *p < 0.05, ***p < 0.005.

The treatment of BMMC with a mixture of the sub‐lethal dose of NT157 and JQ1 (2.5 and 5 μM, respectively) synergistically reduced the viability of the four BMMC lines used in this study by >60% (Figure 1A).

Given these results, we decided to employ a mixture of these two inhibitors at a final concentration of 2.5 μM of NT157 and 5 μM of JQ1 for the rest of the studies. At this concentration, the mixture (designated BRIRi) was cytotoxic to the four BMMC (Figure 1A) and five additional melanoma cell lines (Figure S2).

In subsequent experiments, we examined the growth curve over time of four BMMC lines that were subjected to BRIRi. Control BMMCs were treated with vehicle DMSO. Viability was measured every 24 h up to 96 h. Figure 1B shows that BRIRi reduced cell viability and proliferation over time.

BRIRi is not cytotoxic to human dermal fibroblasts, but it kills about 15% of human astrocytes (Figure 1C). The viability of BRIRi‐treated BECs increased by 30% (Figure 1C).

The viability of BRIRi‐treated microglia was reduced by 25% (Figure 1C). In a recent study, we showed that BMMC‐associated microglia may exert both pro‐metastatic functions as well as anti‐metastatic functions.^17^ Whereas the pro‐metastatic functions were mediated by JunB^hi^ cells (JunB‐overexpressing microglia), the anti‐metastatic functions were enabled by JunB^lo^ cells (JunB‐knocked‐down microglia).^17^ In view of this dichotomy, we asked if these microglia subpopulations differ in susceptibility to the cytotoxic function of BRIRi. Figure 1C shows that BRIRi induced cytotoxicity of 46% in the JunB^hi^ cells, while no change was observed in JunB^lo^ cells.

These data demonstrate that the BRD4 and IRS2 inhibitors can target melanoma cells and melanoma‐promoting non‐cancerous microenvironmental cells in vitro while being inert towards non‐cancerous brain cells.

### 3.2 BRD4 and IRS2 inhibitors modify cell cycle distribution and induce programmed cell death of BMMC

BRIRi‐treated BMMCs were analyzed for cell cycle distribution. Cell cycle arrest was induced by the inhibitors in the four BMMC lines. However, the response of the cell lines was heterogeneous. While 26.5% and 21.6% of BRIRi‐treated YDFR.CB3 and DP.CB2 cells, respectively, were in SubG1 phase, only 9.3% of M16.CB3 and 7.2% of M12.CB3 were arrested in the SubG1 phase.

**FIGURE 2:**
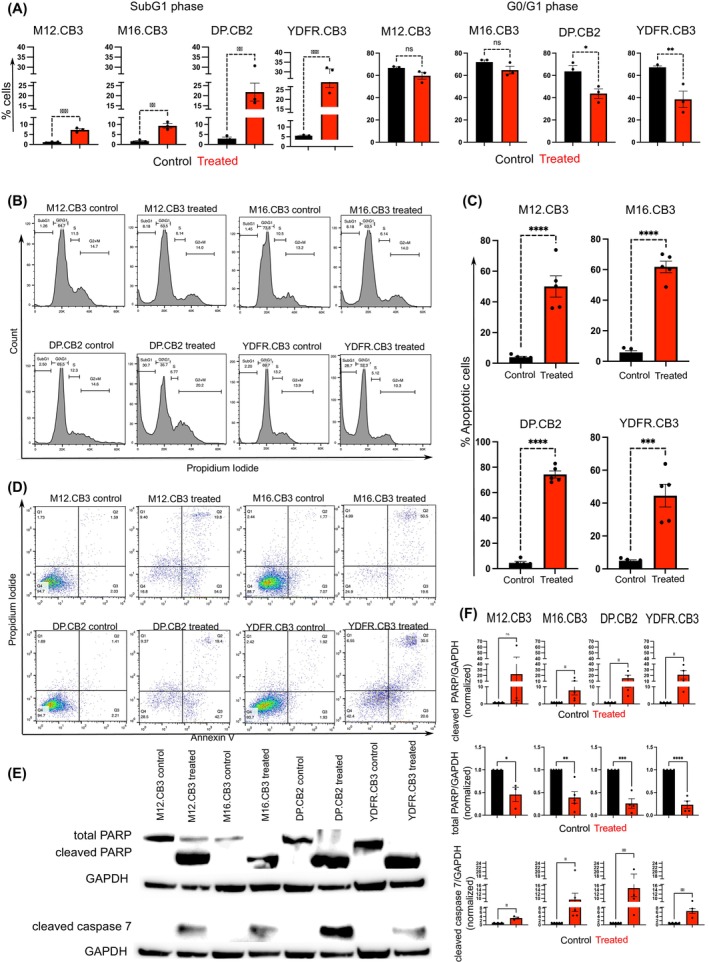
BRD4 and IRS2 inhibitors induce cell cycle arrest and cell death. (A) Cell cycle analysis was performed using flow cytometry to determine the percentage of cells in different phases following treatment with BRIRi. The percentages of cells in the SubG1 and *G*_0_/*G*_1_ phases of the cell cycle were calculated and represented as mean ± SEM of three independent experiments. Significance was evaluated using the Student's *t* test. *p < 0.05, **p < 0.01, ***p < 0.005. (B) Representative images of cell cycle arrest from one of three independent experiments are shown. (C, D) The mode of cell death induced by the BRIRi was evaluated using Annexin V—PI staining. Starvation medium (0.5% FCS) with 0.001% DMSO was used as a control. (C) The percentage of cells that underwent apoptosis was calculated and represented as mean ± SEM of five independent experiments. Significance was calculated using the Student's *t* test. ***p < 0.005, ****p < 0.001. (D) Representative images of cell death in one of five independent experiments are shown. Q1: Necrosis, Q2: Late apoptosis, Q3: Early apoptosis, and Q4: Live cells. (E) Western blot analysis for the expression of total PARP, cleaved PARP, and cleaved caspase 7. GAPDH was used as a loading control. Representative blots of one of at least three independent experiments are shown. (F) The expression of total PARP, cleaved PARP, and cleaved caspase 7 was quantified using ImageJ. Results are shown as mean ± SEM of at least three independent experiments. Significance was evaluated using the Student's *t* test. *p < 0.05, **p < 0.01, ***p < 0.005, ****p < 0.001.

Associated with the increase in the percentage of cells in the SubG1 phase, a concomitant decrease in the percentage of cells in the G0/G1 phase was seen in DP.CB2 and YDFR.CB3 (Figure 2A,B). This result indicates that cells arrested at the sub‐G1 phase likely have undergone DNA fragmentation and apoptosis.^28^

As shown in Figure 2C,D, inhibitors markedly triggered programmed cell death in all four BMMC lines. At least 50% of cells in the four BMMC lines underwent apoptotic cell death. The generation of apoptosis in BRIRi‐treated BMMC is associated with reduced cell viability of such cells (Figure 1A) and with an increased proportion of cells in the SubG1 phase (Figure 2A). The upregulation of cleaved caspase 7 and cleaved PARP proteins in BRIRi‐treated BMMC (Figure 2E,F) is another manifestation of apoptosis in these cells.

### 3.3 BRD4 and IRS2 inhibitors lessen the malignant phenotype of BMMC

Based on the fact that BRIRi mediates the cytotoxicity of BMMC, we examined the impact of the inhibitors on other phenotypic properties of BMMC lines.

**FIGURE 3:**
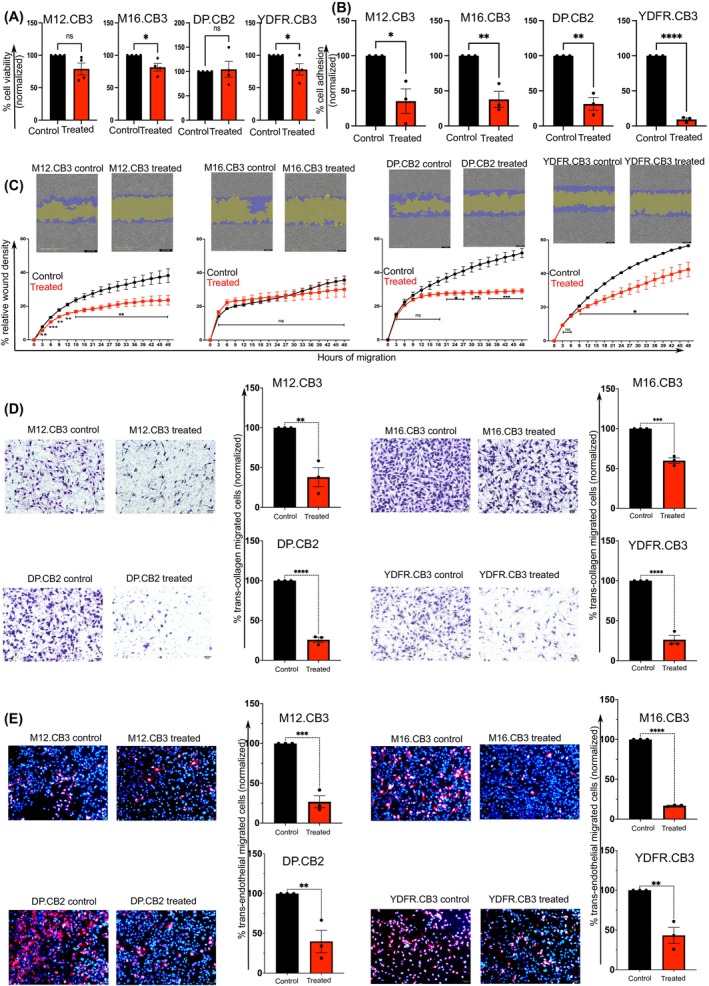
BRD4 and IRS2 inhibitors abrogate BMMC malignancy phenotype. (A) BMMC lines were pretreated with BRIRi for 3 h, starved for 24 h, and then cell viability was measured using the XTT assay. Results are shown as mean ± SEM of four independent experiments. (B) The percentage of melanoma cells treated with BRIRi that adhered to the brain endothelium was calculated and shown as mean ± SEM of three independent experiments. (C) The rate of migration of BRIRi‐treated cells in a wound healing assay was calculated using the Incucyte assay and shown as mean ± SEM of three independent experiments. Representative images of wound closure from one of three independent experiments are shown. (D) The percentage of BRIRi‐treated cells that transmigrated the collagen layer was calculated and shown as mean ± SEM of three independent experiments. Representative images of trans‐collagen migrating cells are shown. (E) The percentage of BRIRi‐treated cells that transmigrated the brain endothelium was calculated and shown as mean ± SEM of three independent experiments. Representative images of trans‐endothelial migrating cells are shown. (A‐E) In all the above experiments, a starvation medium (0.5% FCS) with 0.001% DMSO served as a control. Significance was evaluated using the Student's *t* test. *p < 0.05, **p < 0.01, ***p < 0.005, ****p < 0.001.

Figure 3A demonstrates a heterogeneous response of the BMMC cells to the influence of the inhibitors on cell viability. Whereas treating the cells for 3 h did not impact the cell viability in two cell lines, a 20% reduction in cell viability was measured in the other two lines.

Gandalovičová et al. coined migrastatics for drugs that block cancer cell invasion, migration, and metastasis.^29^ We chose 3 h of inhibitor treatment to study whether the BRIRi possesses migrastatics properties because 3 h of BRIRi treatment was sufficient to induce transcriptomic changes in BMMCs.

In the next set of experiments, we assessed the migrastatic properties of BRIRi, if any.

#### 3.3.1 Adhesion to brain endothelium

Cancer cell adhesion to the endothelium is one of the most critical steps in metastasis.^30^ We assessed, therefore, the ability of the inhibitors to block the adhesion of BMMC to BECs. As shown in Figure 3B, BRIRi treatment reduced BMMC adhesion to brain endothelium by approximately 70% in three out of four BMMC cell lines. Its effect was the highest in YDFR.CB3 cells, whereby it inhibited approximately 90% of the adhesion capacity.

#### 3.3.2 Wound healing

Wound healing assays indicated that BRIRi reduced the migration (relative wound density) of three of four BMMC lines (Figure 3C), but no change was observed in one BMMC line.

#### 3.3.3 Migration and trans‐endothelial migration

Trans‐collagen invasion and trans‐endothelial invasion assays using trans‐wells recapitulate, in vivo, cancer cell invasion through extracellular matrix and endothelium. As illustrated in Figure 3D, BRIRi treatment reduced collagen invasion of all BMMC lines but to different degrees ranging from 40% to 75% inhibition.

Similarly, the inhibitors significantly reduced the capability of BMMC lines to traverse the brain endothelium. Less than 43.2% of treated BMMC retained their ability to penetrate the brain endothelium (Figure 3E).

### 3.4 Normalizing the immunosuppressive microenvironment created by BMMC with BRD4 and IRS2 inhibitors

The interactions between cancer and immune cells can create an immunosuppressive microenvironment through various mechanisms, rendering immune cells unresponsive to the tumor.^31^ This shields the tumor from immune responses, thereby facilitating tumor growth and reducing the effectiveness of immunotherapy.^31^ To determine if BRD4 and IRS2 inhibitors can modulate the BMMCs immunosuppressive cancer microenvironment into an immunocompetent one, we measured the expression of several key tumor‐related immune molecules at the transcriptional and translational levels.

**FIGURE 4:**
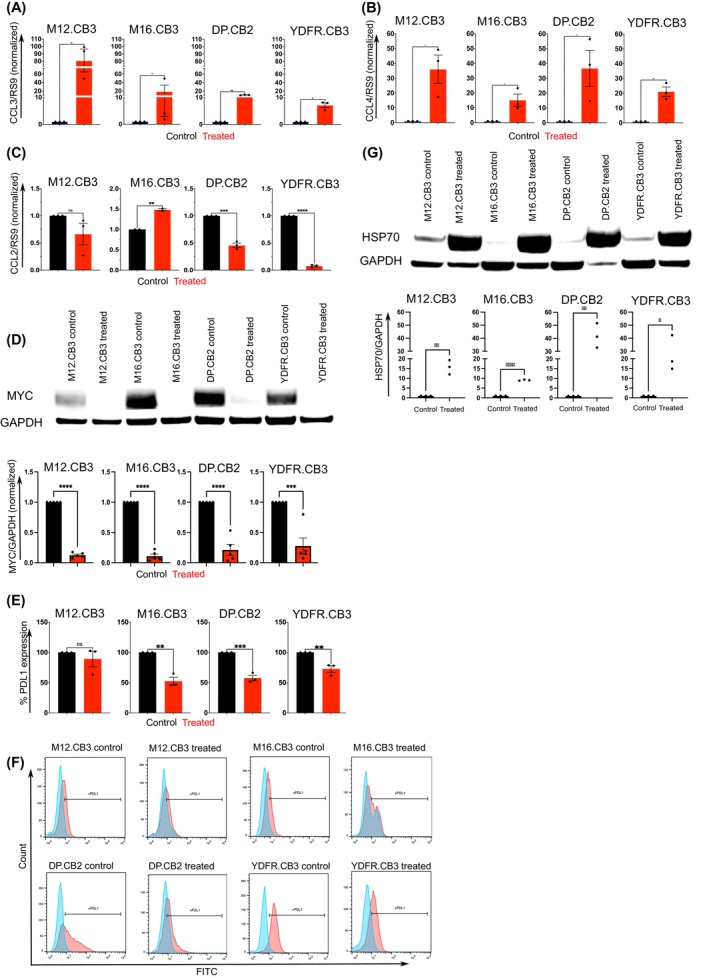
BRD4 and IRS2 inhibitors transform the immunosuppressive phenotype of melanoma. (A–C) mRNA expression of CCL3 (A), CCL4 (B), and CCL2 (C) was quantified in BMMC treated with BRIRi for 3 h. Data are shown as mean ± SEM of three independent experiments. Significance was evaluated using the Student's *t* test. *p < 0.05, **p < 0.01, ***p < 0.005, ****p < 0.001. (D) Western blot analysis of the expression of MYC in cells treated with BRIRi for 24 h. GAPDH was used as a loading control. MYC expression was quantified using ImageJ. Data are shown as mean ± SEM of one of five independent experiments. Representative blots of one of at least five independent experiments are shown. (E–F) The expression of the cell surface marker PD‐L1 was measured by flow cytometry in BMMC, which was pretreated with BRIRi and starved for 24 h. (E) Data are shown as mean ± SEM of one of three independent experiments. (F) Representative histograms from one of three independent experiments are shown. (G) Western blot analysis of the expression of HSP70 in cells treated with BRIRi for 24 h. GAPDH was used as a loading control. Data are shown as mean ± SEM of one of three independent experiments. Representative blots of one of at least three independent experiments are shown. (A–G) Significance was evaluated using the Student's *t* test. *p < 0.05, **p < 0.01, ***p < 0.005, ****p < 0.001.

Since melanoma cells express several chemokines, such as CCL2, CCL3, and CCL4,^32^ playing significant functional roles in the immune TME, we measured the effect of BRIRi on the expression of these chemokines at the transcriptome level. The inhibitors upregulated CCL3 expression in three of four BMMC lines (Figure 4A) and CCL4 expression in all four BMMC lines (Figure 4B). The increased expression of CCL3 and CCL4 could effectively recruit CD8^+^ T cells.^32^

CCL2 is a crucial chemokine that promotes tumorigenesis and an immune‐compromised tumor microenvironment.^33^ Inhibitors decreased CCL2 expression in DP.CB2 and YDFR.CB3 cell lines by 2 and 10 folds, respectively. However, the treatment increased CCL2 expression in M16.CB3 by ~1.5 folds, and no change was observed in M12.CB3 (Figure 4C).

MYC supports the immunosuppressive microenvironment, and its suppression can normalize this effect.^34^, ^35^ In addition, low MYC expression is a biomarker for successful immune checkpoint therapy.^35^ Measuring the expression of the MYC protein in BRIRi‐treated BMMC indicated that the treatment reduced MYC expression in all four BMMC lines (Figure 4D).

In view of the fact that MYC regulates PD‐L1,^36^ a molecule used by tumors to evade T‐cell killing,^37^ we examined the proteomic expression of PD‐L1. The results demonstrated that the inhibitors significantly reduced the expression of PD‐L1 in three BMMC lines (Figure 4E,F).

Heat Shock Protein 70 (HSP70) expression in tumor cells renders them more vulnerable to lysis by Natural Killer (NK) cells.^38^ Therefore, we measured the effects of BRIRi on the expression of the HSP70 protein by BMMC and found that the BRIRi treatment upregulated HSP70 in four BMMC lines (Figure 4G).

Overall, these results suggested that BRD4 and IRS2 inhibitors might be used at the pre‐clinical and clinical level to transform the immunosuppressive microenvironment of BMMC into a more immunocompetent one.

### 3.5 BRD4 and IRS2 inhibitors re‐program the transcriptome of BMMC

**FIGURE 5:**
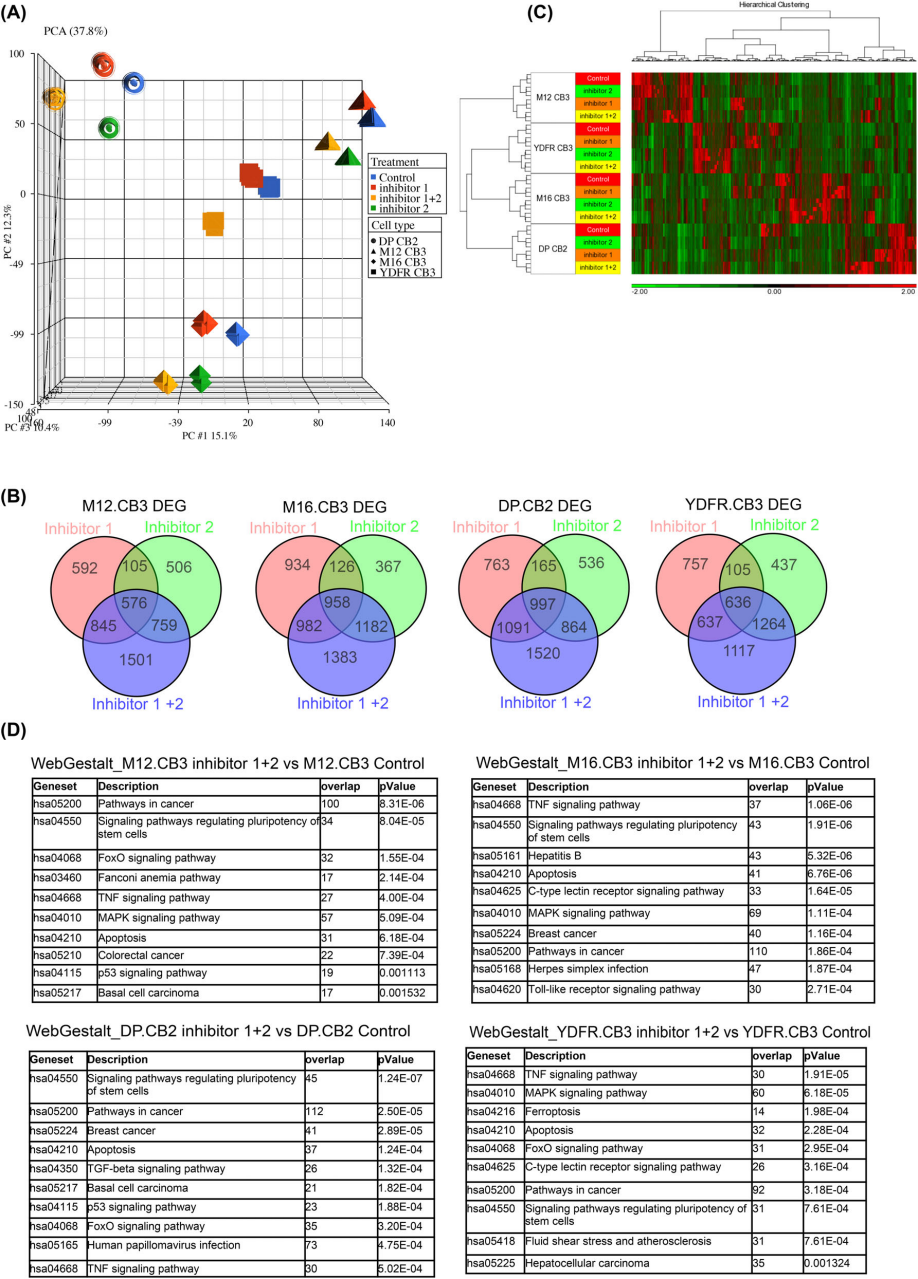
BRD4 and IRS2 inhibitors alter the transcriptomic landscape of BMMC. (A) Principal component analysis (PCA) of bulk RNA‐seq data (DMSO‐treated cells, BRD4 inhibitor‐treated cells, IRS2 inhibitor‐treated cells, and BRIRi‐treated cells) of four BMMC lines shows a distinct difference between DMSO‐treated cells and BRIRi‐treated cells. (B) Venn diagrams depict the total number of genes that are differentially expressed post different inhibitor treatments in four BMMCs. (C) Clustered analysis of DMSO‐treated cells, BRD4i‐treated cells, IRS2 inhibitor‐treated cells, and BRIRi‐treated cells from the RNA‐seq data. (D) Tables of the top 10 enriched functions of differentially expressed genes derived from the RNA‐seq data using DAVID. (A–D) inhibitor 1 refers to NT157 (IRS2 inhibitor), inhibitor 2 refers to JQ1 (BRD4i).

We performed a bulk RNA‐seq analysis of BRIRi treated BMMC to identify the signaling pathways controlled by BRIRi. The principal component analysis of the transcriptomic data revealed that treatment with the BRD4 inhibitor alone, the IRS2 inhibitor alone, or the mixture of both induces transcriptomic variations across the treatment conditions and across four cell lines (Figure 5A).

When subjected to both BRD4 and IRS2 inhibitors, as opposed to administering the inhibitors individually, a greater number of gene expressions were modified in M12, CB3, M16, CB3, and YDFR, CB3 (Figure 5B).

The cluster analysis of treated and untreated cell lines corroborates the variations among four BMMC lines and illustrates the presence of inter‐tumor heterogeneity, characterized by unique gene expression patterns for each cell type (Figure 5C).

Based on RNA‐seq data, we performed a function enrichment analysis on differentially expressed genes. Figure 5D shows that in spite of inter‐tumor heterogeneity, the treatment with BRIRi altered, in all four BMMC lines, the expression of genes belonging to three signaling pathways: the TNF signaling pathway, the pathway regulating pluripotency of stem cells, and the apoptotic pathway, which were commonly overrepresented in all four melanomas.

## 4 DISCUSSION

In addition to innate and adaptive anticancer immunity, several additional factors exert anticancer functions. These cancer‐inhibitory functions are mediated by normal cells, such as fibroblasts^39^ or anti‐microbial peptides.^40^

Investigating mechanisms that could keep dormant metastatic cancer cells^15^ in check, we discovered that pulmonary endothelial and epithelial cells produce the beta subunit of hemoglobin (HBB) and that this protein is cytotoxic to lung‐invading neuroblastoma cells. An HBB‐derived peptide (METOX) exhibited significant anti‐metastatic functions in vitro and in vivo.^8^ Subsequent studies performed in another lab indicated that HBB regulates the proliferation of lung cancer cells and may serve as a diagnostic biomarker for this malignancy.^41^ In another study, the authors combined METOX with a small‐molecule catenin inhibitor to generate a supramolecular nanomedicine with enhanced tumor targeting.^42^

Searching for brain‐derived molecules with the capacity to induce cytotoxicity of brain‐metastasizing melanoma cells (BMMC), we discovered that an α/β hemoglobin dimer induces apoptosis/necrosis of BMMC and downregulates three proteins: BRD4, GAB2, and IRS2, which are crucial components of cell viability and sustainability.^43^, ^44^, ^45^

Previous studies indicated that the expression of hemoglobin components is not restricted to erythrocytes and that a variety of cells, including alveolar cells,^8^ brain cells^9^, ^46^ and others^47^ express hemoglobin and/or its subunits.

These and other reports^48^, ^49^ show that hemoglobin and hemoglobin‐derived molecules exhibit anti‐cancer functions similar to “classical” antimicrobial peptides and proteins. The translation of these studies to the clinic may provide novel anti‐cancer treatment modalities.

The present study establishes proof of concept that innate anticancer factors may serve as “pathfinders” that lead to the identification of targetable therapy targets on cancer cells.

To explore the potential of BRD4, GAB2, and IRS2 as therapeutic targets for melanoma brain metastasis, we evaluated the anti‐melanoma functions of small‐molecule inhibitors of these proteins.

The GAB2 inhibitor imperatorin displayed only limited cytotoxicity of BMMC cell lines and, therefore, was not further studied. However, we showed that the synergistic inhibition of BRD4 by the JQ1 inhibitor and of IRS2 by the NT157 inhibitor exerted a significant anti‐melanoma cell impact.

Whereas, a single treatment of BMMC by the JQ1/NT157 combination significantly reduced viability over time, the inhibitors were not cytotoxic to some noncancerous brain cells such as brain‐endothelial cells or to anti‐metastatic microglia cells. On the other hand, BRIRi exhibited a significant toxicity to astrocytes. Since astrocytes may support melanoma brain metastasis,^50^ their suppression may be beneficial.

The BRIRi‐mediated growth arrest of BMMC was observed at the molecular and cellular levels. Cell cycle distribution analysis demonstrated a subG1 phase arrest and extensive DNA fragmentation as characteristics of apoptosis. A sharp increase in the expression of cleaved caspase 7 and cleaved PARP validated that BRIRi induced apoptosis of BMMC.

BRIRi also functioned as an antimetastatic agent by reducing BMMC adhesion to BECs and BMMC migration and invasion capabilities.

BRIRi can generate anti‐melanoma immunomodulatory functions by upregulating CD8^+^ T‐cell‐recruiting CCL3 and CCL4 expressed by melanoma cells^32^ and by downregulating the expression of the immunosuppressive chemokine CCL2.^33^ The inhibitors significantly downregulated the expression of MYC, which, in addition to its tumorigenesis‐promoting activity, also enables cancer cells to evade anti‐cancer immune responses.^51^ In line with the observation that MYC controls PD‐L1 expression,^36^ we also found that the BRIRi downregulated PD‐L1 expression on BMMC. A recent review^52^ described various strategies and chemicals to downregulate PD‐L1 expression in cancer cells. In view of the finding that the BRD4/IRS2 inhibitor cocktail downregulates the expression of PD‐L1 in melanoma cells, we propose that these inhibitors may represent an additional approach to reduce the expression of PD‐L1 in cancer cells.

Taken together, we have proven the concept that innate anticancer resistance factors may serve as pathfinders for the detection of therapy targets on cancer cells.

An additional issue should be raised in connection with the present study and many others in cancer biology. Inter‐tumor heterogeneity is a significant challenge confronting oncologists and basic cancer biologists. This heterogeneity is manifested, inter alia, by distinct phenotypic profiles of tumors with the same histology (e.g., melanoma) and varied responses to stimuli (growth factors, chemical or mechanical stress, drugs, etc.). In the clinic, it is one of the major reasons for patients' differential responses to a given therapy modality.

The present study, as well as previous ones from our lab, clearly indicated that multiple cell lines of brain‐metastasizing melanomas originating from different patients differed in their transcriptome, proteome, response to signals, and response to genetic manipulations.^20^, ^53^

Given this heterogeneity, one should be cautious of generalizations when interpreting the results of experiments employing a single or a small sample size.

Transcriptomic analysis of four BRIRi‐treated BMMC cell lines revealed distinct responses to this treatment. However, clustered examination of the transcriptome data identified three pathways (TNF signaling, apoptosis, and pathways regulating pluripotency of stem cells) that were equally influenced in the four BMMC lines, thereby underscoring the therapeutic potential across various melanoma subtypes. These findings underline the therapeutic potential of the combination of BRD4 and IRS2 inhibitors with respect to melanoma therapy.

## AUTHOR CONTRIBUTIONS

**Maharrish Chelladurai:** Conceptualization; investigation; writing – original draft; validation; visualization; writing – review and editing; formal analysis. **Orit Sagi‐Assif:** Investigation. **Shlomit Ben‐Menachem:** Investigation. **Tsipi Meshel:** Investigation. **Metsada Pasmanik‐Chor:** Software; data curation. **Sivan Izraely:** Writing – review and editing; project administration. **Dave S. B. Hoon:** Resources; writing – review and editing; funding acquisition. **Isaac P. Witz:** Conceptualization; funding acquisition; writing – original draft; writing – review and editing; supervision.

## CONFLICT OF INTEREST STATEMENT

The authors have declared that no competing interests exist.

## Supporting information


**DATA S1.** Supporting Information.

## Data Availability

The RNAseq data is deposited in the GEO database under accession number GSE278118. Other data supporting this study's findings are available from the corresponding author upon request.
