# Supplementary material for: A heterodimer of hemoglobin identifies theranostic targets on brain‐metastasizing melanoma cells
Source: Int J Cancer. 2025 Apr 26;157(4):773–87. doi: 10.1002/ijc.35458 (PMC12178103; doi:10.1002/ijc.35458)
Supplement: Supplementary file 1 — DATA S1. Supporting Information. [file IJC-157-773-s001.pdf]

A heterodimer of hemoglobin identifies theranostic targets on brain-metastasizing melanoma cells

Maharrish Chelladurai, Orit Sagi-Assif, Shlomit Ben-Menachem, Tsipi Meshel, Metsada Pasmanik-Chor, Sivan Izraely, Dave S. B. Hoon, Isaac P. Witz

Table of contents:

1. Supplementary Figure 1
2. Supplementary Figure 2
3. Supplementary Table 1

Supplementary Figure S1

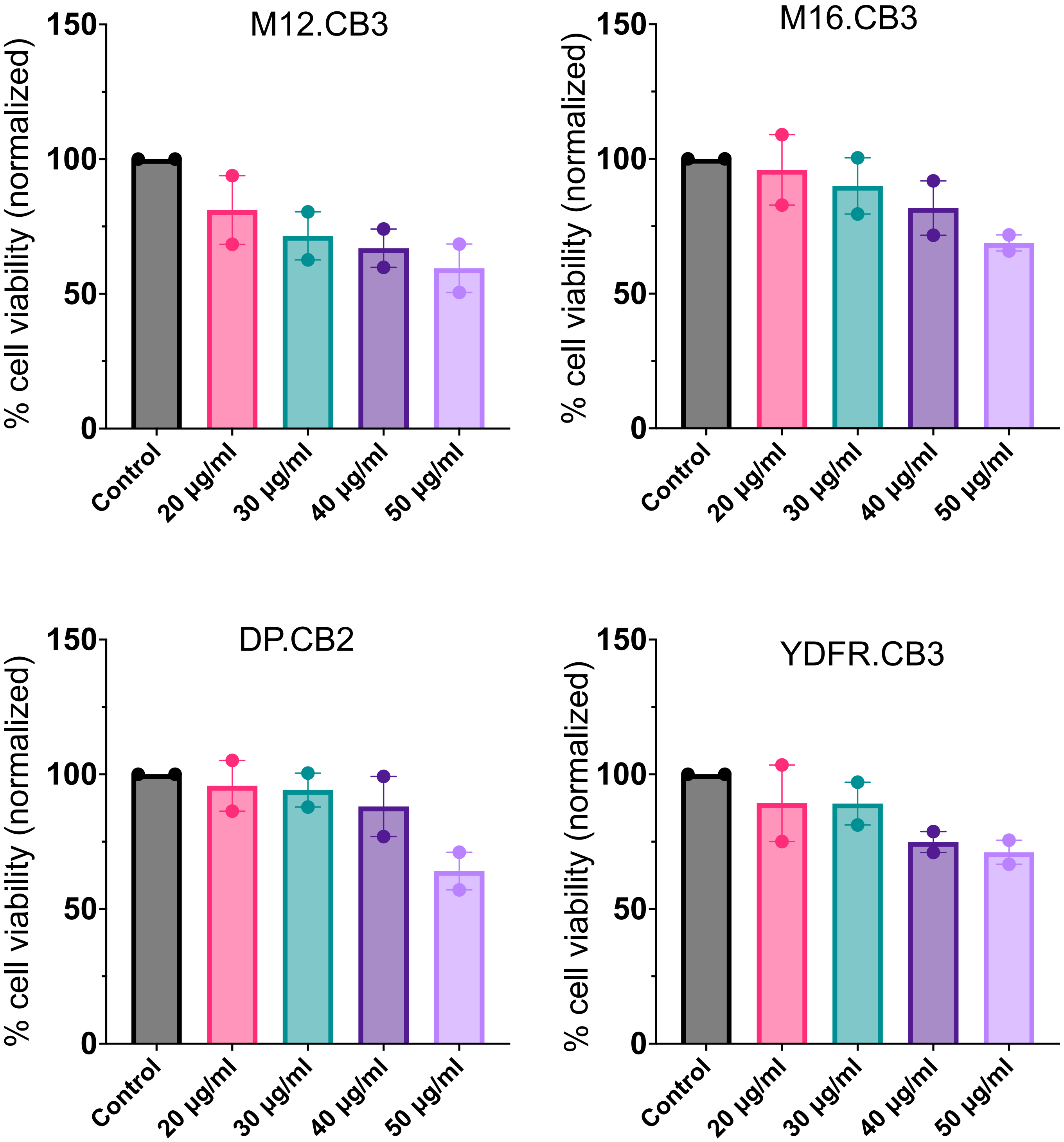

Figure S1. The cytotoxicity of imperatorin (GAB2i) was evaluated on four BMMC lines after treatment for 24 hours. Starvation medium (0.5% FCS) with 0.001% DMSO served as a control. Results are shown as mean ± SEM of two independent experiments.

Supplementary Figure S2

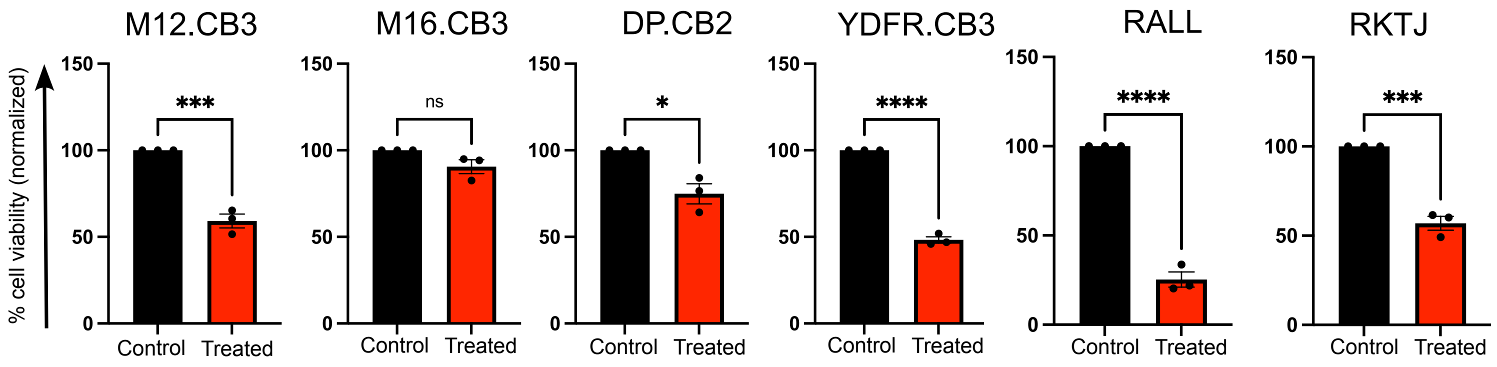

Figure S2. Cytotoxicity of a combination of JQ1 (2.5 [illegible]M) and NT157 (5[illegible]M) in six melanoma cell lines was evaluated after treating cells for 24 hours. Starvation medium (0.5% FCS) with 0.001% DMSO served as a control. Results are shown as mean ± SEM of three independent experiments. Significance was evaluated using the student's t-test. *p<0.05, ***p<0.005, ****p<0.001.

Supplementary Table 1: Sequencing Coverage and Quality Statistics of RNA sequencing (RNA-seq)

Sample ID	Total number of sequenced reads	Total number of uniquely mapped reads[a]	RNA integrity number (RIN)	Ratio of all reads aligned to rRNA regions to total uniquely mapped reads (rRNA rate)	Ratio of exon-mapped reads to total uniquely mapped reads (Expression Profile Efficiency)	Total number of detected genes with reads ≥1[b]
DP-CB2-1-2-I_S2	31749753	26555933	9.8	1.1955804	86.01%	34880
DP-CB2-1-2-II_S3	31771292	26549901	9.8	1.19666329	86.08%	34881
DP-CB2-1-2-III_S4	31773166	26573441	9.6	1.19567376	85.61%	35096
DP-CB2-Control-I_S9	32476000	28436235	9.7	1.14206399	85.70%	32076
DP-CB2-Control-II_S10	33828638	29535401	9.6	1.14535902	85.81%	32030
DP-CB2-Control-III_S11	31623472	27673293	9.3	1.14274337	85.72%	31984
DP-CB2-inhibitor-1-I_S12	29717282	25121263	9.6	1.18295334	85.97%	34219
DP-CB2-inhibitor-1-II_S13	30322102	25619348	9.5	1.18356259	86.01%	34352
DP-CB2-inhibitor-1-III_S14	29080853	24544242	9.7	1.18483402	86.04%	34023
DP-CB2-inhibitor-2-I_S15	33024046	28634655	9.7	1.15328947	86.03%	32246
DP-CB2-inhibitor-2-II_S16	35369969	30670097	9.6	1.15323955	85.61%	32660
DP-CB2-inhibitor-2-III_S1	33697632	29221172	9.7	1.15319235	84.98%	32774
M12-CB3-1plus2-I_S10	31806808	26472449	8.6	1.20150606	83.64%	33199
M12-CB3-1plus2-II_S11	33435803	27817628	9.7	1.20196456	83.66%	33267
M12-CB3-1plus2-III_S12	26940824	22368761	9.6	1.204395	83.53%	34721
M12-CB3-Control-I_S1	28687312	25031707	9.7	1.14603898	85.82%	30735
M12-CB3-Control-II_S2	34236010	29936283	9.6	1.14362929	86.63%	31065

M12-CB3-Control-III_S3	33737178	29482711	9.4	1.14430379	85.62%	31180
M12-CB3-inhibitor-1-I_S4	31917908	26980292	9.6	1.18300825	84.62%	32466
M12-CB3-inhibitor-1-II_S5	31962294	26971771	9.7	1.18502764	85.03%	32177
M12-CB3-inhibitor-1-III_S6	33311543	28115292	9.7	1.18481939	84.59%	32402
M12-CB3-inhibitor-2-I_S7	28257737	24466504	9.6	1.15495606	84.51%	31554
M12-CB3-inhibitor-2-II_S8	30186182	26139002	9.9	1.154833	84.55%	31731
M12-CB3-inhibitor-2-III_S9	32079403	27743615	9.8	1.15628057	84.72%	31697
M16-CB3-1-2-I_S6	33469030	27230873	9.5	1.22908399	84.99%	35359
M16-CB3-1-2-II_S7	30426931	24794863	9.5	1.22714657	85.04%	35177
M16-CB3-1-2-III_S8	26791817	21808442	9.6	1.22850669	84.89%	34636
M16-CB3-Control-I_S13	29870891	25655279	9.5	1.16431753	85.53%	32404
M16-CB3-Control-II_S14	31064133	25727923	9.6	1.20740928	85.38%	32484
M16-CB3-Control-III_S15	30903385	26529742	9.6	1.16485811	85.50%	32567
M16-CB3-inhibitor-1-I_S16	30698287	25553539	9.4	1.20133211	84.89%	33590
M16-CB3-inhibitor-1-II_S1	31063185	25802348	9.6	1.20388986	85.19%	33292
M16-CB3-inhibitor-1-III_S2	34837851	28970260	9.7	1.20253843	85.25%	33370
M16-CB3-inhibitor-2-I_S3	32886259	27945610	9.6	1.17679517	84.56%	33357
M16-CB3-inhibitor-2-II_S4	25557602	21666746	9.5	1.17957731	85.09%	34253
M16-CB3-inhibitor-2-III_S5	24041237	20392227	9.5	1.17894122	85.07%	34148
YDFR-CB3-1-2-I_S14	36008278	30581615	9.7	1.17744854	84.75%	32827
YDFR-CB3-1-2-II_S15	32988250	27973021	9.7	1.17928807	84.23%	32723
YDFR-CB3-1-2-III_S16	26523412	22544706	9.8	1.17648072	83.96%	34115
YDFR-CB3-Control-I_S5	33729065	29382745	9.8	1.14792083	84.93%	31752
YDFR-CB3-Control-III_S7	31185735	27162033	9.8	1.148137	85.63%	31260

YDFR-CB3-inhibitor-1-I_S8	31100020	26833334	9.9	1.15900693	84.43%	32328
YDFR-CB3-inhibitor-1-II_S9	32132458	27665512	9.8	1.16146262	84.14%	32462
YDFR-CB3-inhibitor-1-III_S10	33462053	28837057	9.8	1.16038377	84.33%	32329
YDFR-CB3-inhibitor-2-I_S11	30787139	26545122	9.8	1.15980401	85.38%	31975
YDFR-CB3-inhibitor-2-II_S12	22863796	19727047	9.8	1.15900753	84.63%	33187
YDFR-CB3-inhibitor-2-III_S13	31456241	27143528	9.7	1.1588855	84.80%	32195

[a]GENCODE RRID:SCR_004463 comprehensive gene annotation reference set (version 19) using the STAR aligner (version 2.4.2a) RRID:SCR_004463 was used for reference.

[b]A higher minimum coverage threshold is permitted.
